# From Nerves to Nodules: A Case-Based Review of the Progression of Pure Neuritic Leprosy to Lepromatous Leprosy With Erythema Nodosum Leprosum

**DOI:** 10.7759/cureus.98463

**Published:** 2025-12-04

**Authors:** Shini Choubey, Hemanta K Kar, Debasmita Behera

**Affiliations:** 1 Dermatology, Venereology and Leprosy, Kalinga Institute of Medical Sciences, Bhubaneswar, IND

**Keywords:** erythema nodosum leprosum, infectious and tropical diseases, lepromatous leprosy, multidrug therapy (mdt), pure neuritic leprosy

## Abstract

Leprosy, caused by *Mycobacterium leprae*, affects the skin and peripheral nerves. Pure neuritic leprosy (PNL) is a rare subtype, characterized by nerve involvement without skin lesions. We report a rare case of PNL progressing to lepromatous leprosy (LL) with erythema nodosum leprosum (ENL), with only one such case previously reported in the literature and reviewed herein. A 35-year-old male diagnosed with PNL on sural nerve biopsy showing acid-fast bacilli (3+) completed multidrug therapy for 12 months. Nine months later, he developed painful erythematous nodules and sensory-motor deficits. Slit-skin smear (bacteriological index 4+) and biopsy confirmed LL with ENL. He was treated with monthly pulses of rifampicin, minocycline, and moxifloxacin, along with thalidomide and corticosteroids. Significant improvement occurred after six months. This case illustrates the intricate clinical challenges associated with leprosy, particularly how PNL can progress into more severe forms such as LL. While Type 1 reactions are documented in PNL, only a single instance of a Type 2 reaction has been recorded in PNL. Persistent bacterial load, immune modulation, host factors, and drug resistance can all contribute to this shift, necessitating vigilant monitoring even after treatment completion. This case underscores the rare transformation of PNL to LL, possibly due to persistent bacilli and reduced cell-mediated immunity, emphasizing the importance of early diagnosis, complete therapy, and careful follow-up.

## Introduction

Leprosy, a chronic infectious disease caused by *Mycobacterium leprae*, manifests across a clinical spectrum, with nerve involvement being a hallmark. Pure neuritic leprosy (PNL) is a rare subtype, particularly seen in the Indian subcontinent (2.17-5.3%), characterized by isolated peripheral nerve damage without skin lesions, often delaying diagnosis and treatment [1]. PNL may gradually involve multiple nerves and affect the surrounding skin supplied by the involved nerves, a phenomenon known as secondary neuritic leprosy. This typically occurs in the borderline spectrum of the disease (BT to BB, rarely BL) when the body’s cell-mediated immunity (CMI) gradually deteriorates due to delayed initiation of anti-leprosy treatment. In PNL, neuritis more commonly manifests as Type 1 reactions (T1Rs) and only rarely as Type 2 reactions (T2Rs) [2]. In this report, we present a rare case of PNL that unexpectedly progressed to lepromatous leprosy (LL), the most severe and disseminated form of leprosy, complicated by erythema nodosum leprosum (ENL), or T2R [3].

ENL lesions are manifestations of T2R, a multisystem, immune-mediated complication of LL and, rarely, BL leprosy. T2R is characterized by the sudden development of tender skin lesions (ENL), neuritis, arthritis, dactylitis, orchitis, lymphadenitis, osteitis, ocular complications, and nephritis, with or without fever or malaise, and histological features consistent with ENL on tissue biopsy. ENL can be the initial manifestation of the disease or occur during the course of treatment. It is considered a neutrophilic, immune complex-mediated reaction, with evidence of complement, protein, and immunoglobulin deposition in the dermis and other tissues.

This article was previously presented as a poster and meeting abstract at the 105th British Association of Dermatologists’ Annual Meeting in Glasgow on June 1, 2025.

## Case presentation

History and examination

A 35-year-old male from a leprosy-endemic region of India presented with progressive weakness and loss of sensation in both hands and feet over the past eight months. He was diagnosed with PNL in August 2022, confirmed by sural nerve biopsy, which showed acid-fast bacilli (AFB; 3+) and granulomatous inflammation, along with the presence of *M. leprae *within macrophages. Based on this diagnosis, he was started on WHO-recommended multidrug therapy (MDT) with rifampicin, dapsone, and clofazimine for 12 months.

**Figure 1:**
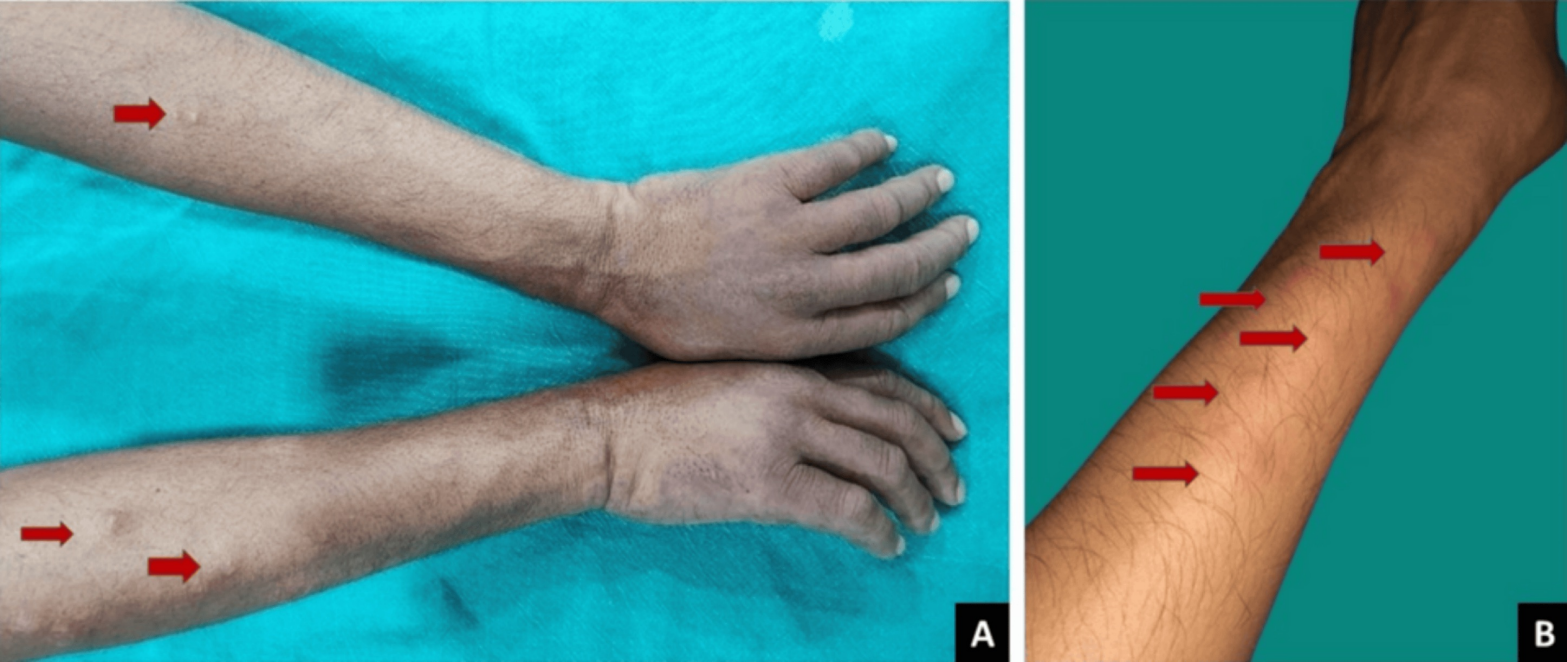
Baseline images (A) Multiple erythematous nodules (red arrows), 0.5-1.5 cm in diameter, present over the extensor aspects of both forearms, along with edema of the dorsum of the hands. (B) Zoomed-in view of erythematous nodules, clinically suggestive of ENL. ENL, erythema nodosum leprosum

During MDT, the patient experienced improvement in neurological symptoms, including sensory loss and motor deficits. The bacteriological index (BI) on skin smear examination also improved, correlating well with the clinical recovery. However, nine months after completing MDT, he developed numerous painful erythematous nodules on his face, trunk, and extremities, accompanied by systemic symptoms including fever, malaise, and joint pain (Figure 1A, 1B).

These lesions were associated with progressive numbness, muscle weakness, and sensory deficits in both hands and feet. There was complete loss of sensation in the distribution of the ulnar, median, and lateral popliteal nerves. Examination revealed enlargement and tenderness of the left ulnar nerve with neuritis, partial ulnar claw hand, and atrophy of the small muscles of the left hand.

Investigations

Slit-skin smear examination was performed from the bilateral earlobes, bilateral eyebrows, left buttock, and nodule, following the standard WHO protocol using Ziehl-Neelsen staining for AFB. This revealed a positive BI of 4+ [1].

**Figure 2:**
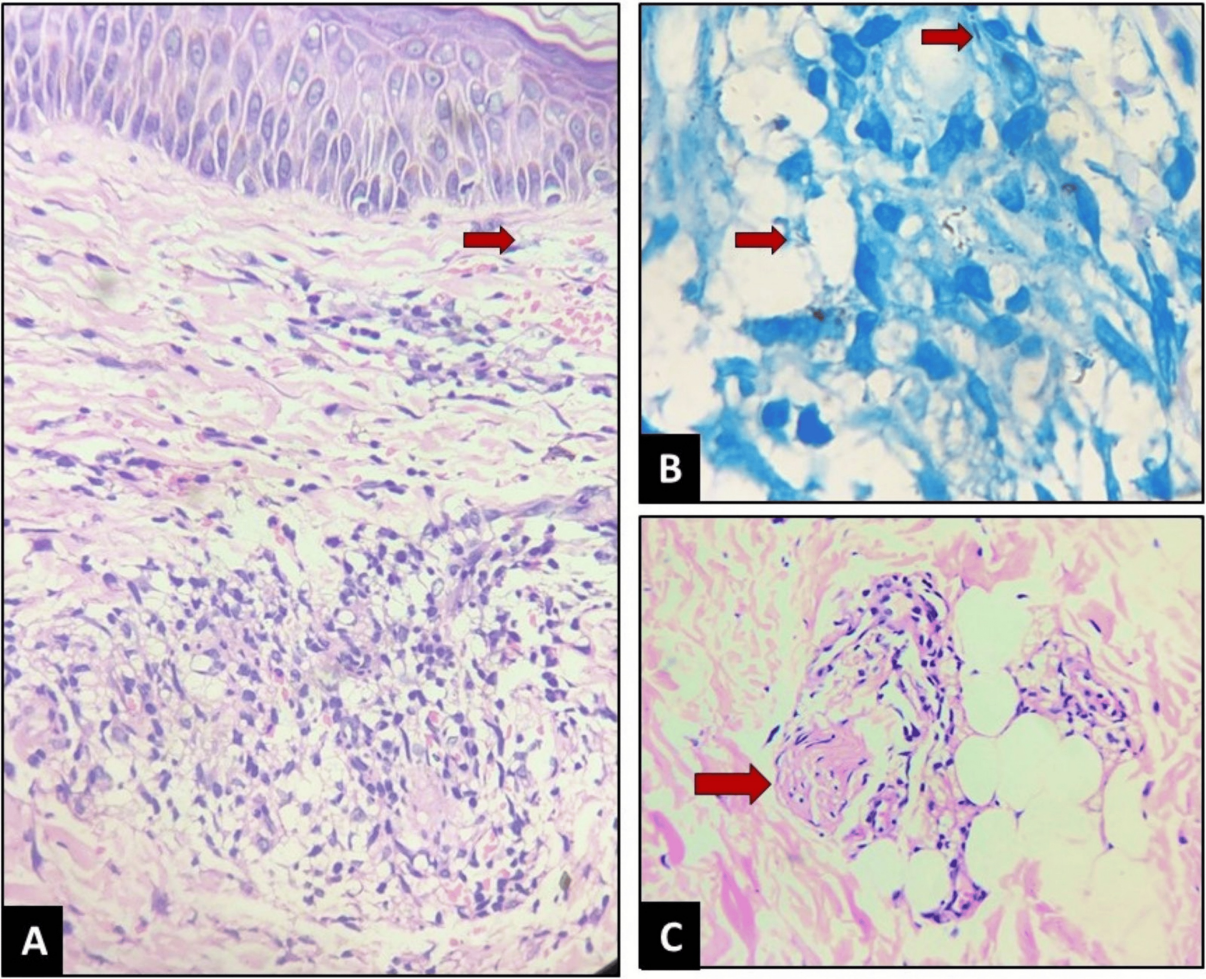
Histopathology images (A) H&E stain, 10×: reticular dermal infiltration of lymphocytes, flattening of the rete ridges, and a prominent Grenz zone (red arrow). The dermis shows inflammatory cell infiltration with foamy macrophages. (B) Lepra stain, 40×: presence of AFB (red arrows) with a BI of 1+. (C) H&E stain, 40×: granular polymorphonuclear infiltration with perineural lymphocytic infiltration and lobar panniculitis (nerve fiber indicated by red arrow). AFB, acid-fast bacilli; BI, bacteriological index

Histopathological examination of the skin revealed positive AFB and showed reticular dermal infiltration of lymphocytes, flattening of the rete ridges, and a prominent Grenz zone. The dermis demonstrated inflammatory cell infiltration with foamy macrophages, consistent with a diagnosis of LL with ENL (Figure 2A-2C).

Management

Three bactericidal drugs were administered in a monthly pulse regimen for 12 months due to the suspected presence of drug-resistant *M. leprae*: rifampicin 600 mg, minocycline 200 mg, and moxifloxacin 400 mg, each given as a once-monthly pulse. For management of ENL, thalidomide was initiated at 300 mg daily for seven days, followed by 200 mg daily for the next month, and then 100 mg daily for the subsequent three months to control the severe ENL reaction. Additionally, oral prednisolone (1 mg/kg body weight) was administered for the initial 12 weeks, with a tapering dose, to treat left ulnar neuritis.

**Figure 3:**
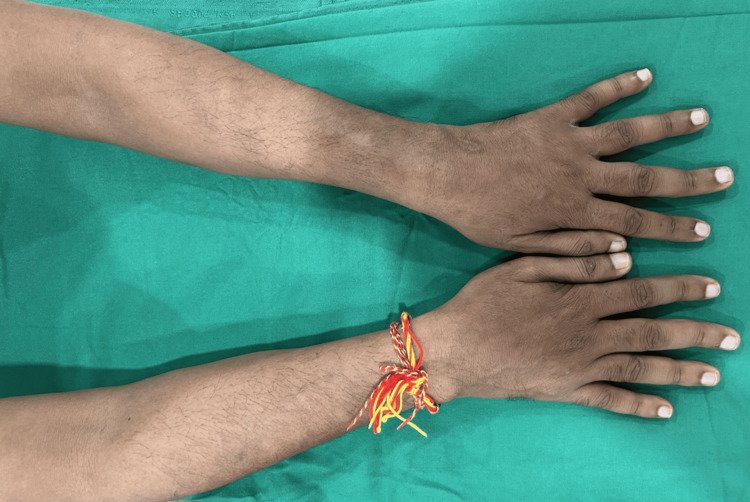
Follow-up image Complete improvement was observed at six months of follow-up, with no evidence of reaction, erythema, edema, or new lesions following monthly pulse therapy with rifampicin (600 mg), minocycline (200 mg), and moxifloxacin (400 mg).

At the six-month follow-up, the patient responded favorably to treatment, with no new ENL lesions or neuritis (Figure 3). Peripheral sensation and mobility of the partial claw hand improved with standard physiotherapy and the use of a claw-hand splint. The patient has been followed for 12 months with no recurrence.

## Discussion

This case illustrates the clinical challenges associated with leprosy, particularly how PNL can progress to more severe forms, such as LL. The onset of ENL further complicates management, necessitating careful treatment strategies. While T1Rs are commonly documented in PNL, only a single instance of a T2R has been previously reported in PNL (Table 1) [4].

**Table 1:** Review of the reported cases of PNL with T2R BI, bacteriological index; MB-MDT, multibacillary multidrug therapy; MDT, multidrug therapy; PNL, pure neuritic leprosy; RMM, rifampicin, minocycline, moxifloxacin; T2R, Type 2 reaction

Case	Age	Gender	Duration after which the reaction occurred	Slit-skin smear	Lab investigations		Management
					PNL	T2R	
Fernandes et al. (2020) [4]	30	Male	Two months after diagnosis and initiation of MDT	Negative	Presence of *Mycobacterium leprae* in sural nerve biopsy; nerve conduction study	Skin biopsy sample for histopathology; BI: negative	PNL: MB-MDT with prednisolone; T2R: thalidomide and steroids; MDT continued
This case	35	Male	Nine months after diagnosis of PNL and initiation of MDT	Baseline: negative; T2R: 4+	Presence of lepra bacilli in sural nerve biopsy: BI: 3+; nerve conduction study	Skin biopsy sample for histopathology: BI: 1+	PNL: MB-MDT prednisolone; T2R: thalidomide and steroids; rifampicin 600 mg, minocycline 200 mg, and moxifloxacin 400 mg (RMM monthly once pulse for 12 months)

Differential diagnosis of PNL is essential due to overlapping symptoms with various neurological conditions. Clinicians must consider several potential alternatives, including Guillain-Barré syndrome, which presents with rapid-onset muscle weakness and sensory alterations. Diabetic neuropathy, characterized by sensory loss and pain primarily in the extremities, is another important consideration [5]. Multiple sclerosis may also mimic PNL, as it can cause diverse neurological symptoms, including motor deficits. Additionally, conditions such as chronic inflammatory demyelinating polyneuropathy, sarcoidosis, and HIV-associated neuropathy can exhibit similar presentations. Infectious causes like vitamin B12 deficiency and Lyme disease may also lead to peripheral neuropathy. Hereditary neuropathies, such as Charcot-Marie-Tooth disease, should be evaluated. Each of these conditions requires thorough clinical assessment and specific diagnostic tests to ensure accurate differentiation, highlighting the importance of an interdisciplinary approach in managing patients suspected of having PNL [6].

Recently, there has been a rise in atypical presentations of leprosy, including the development of skin lesions after peripheral nerve involvement, commonly observed in the borderline spectrum [7]. Drug resistance may contribute to changes in disease spectrum from PNL to LL; however, due to the patient’s economic constraints, a resistance study was not performed.

We hypothesize that drug-resistant *M. leprae* bacilli and/or persisters in the peripheral nerves, even after a complete course of MDT, play a significant role in reactivation or relapse of leprosy by spreading from the nerves to the skin, leading to downgrading toward LL following reduced CMI [8]. ENL usually develops due to rapid bacterial killing following MDT, either during or after treatment, and rarely before treatment. Persisters typically cause relapse after MDT completion due to their multiplication and may also trigger T2Rs. The exact mechanism remains unclear, though reduced CMI likely plays a key role [9]. All such cases require urgent drug-sensitivity testing and immunological evaluation. Lepromin testing could not be performed in this case due to its unavailability in India, preventing assessment of CMI status.

## Conclusions

While T1Rs, with or without skin lesions, are known to occur in PNL, cases with a high bacillary load and low CMI may show downgrading toward the lepromatous spectrum and develop T2Rs, which is a rare phenomenon. This case emphasizes the importance of early diagnosis of all PNL cases, vigilant follow-up, and awareness of potential disease transformations, particularly in endemic areas.
